# National screening for developmental delays and their determinants among Egyptian school age children: A step towards implementing life skills programs

**DOI:** 10.1371/journal.pone.0287315

**Published:** 2023-09-19

**Authors:** Ammal M. Metwally, Maysa S. Nassar, Ebtissam M. Salah El-Din, Ali M. Abdallah, Zeinab Khadr, Marwa W. Abouelnaga, Engy A. Ashaat, Mostafa M. El-Saied, Ahmed M. Elwan, Randa I. Bassiouni, Zeinab M. Monir, Hala Y. Badawy, Eman M. Dewdar, Hazem M. El-Hariri, Ahmed Aboulghate, Carine Hanna, Thanaa M. Rabah, Amira Mohsen, Mona A. Elabd

**Affiliations:** 1 Community Medicine Research Department, Medical Research and Clinical Studies Institute, National Research Centre, Dokki, Cairo, Egypt; 2 Child Health Department, Medical Research and Clinical Studies Institute, National Research Centre, Dokki, Cairo, Egypt; 3 Quantitative Methods Department, Aswan University, Tingar, Egypt; 4 Department of Statistics, Faculty of Economics and Political Science, Cairo University, Giza, Egypt; 5 The Social Research Center of the American University in Cairo, New Cairo, Egypt; 6 Clinical Genetics Dept., Human Genetics and Genome Research Institute, National Research Centre, Dokki, Cairo, Egypt; 7 Child with Special Needs Dept., Medical Research and Clinical Studies Institute, National Research Centre, Dokki, Cairo, Egypt; 8 Disability Prevention General Directorate, Ministry of Health and Population, Cairo, Egypt; Wolaita Sodo University, ETHIOPIA

## Abstract

**Aim:**

This study aimed to estimate the national prevalence of developmental delays (DDs) and their determinants among Egyptian children aged 6 to 12 years. Such estimation is a prerequisite step toward the application of Life Skill Education (LSE) programs that will potentiate children’s future capabilities.

**Methods:**

Vineland Adaptive Behavior Scales” was used as a reliable and diagnostic test for DDs screening during this national cross sectional study. Gross motor (GM), fine motor (FM), daily living skills, communication, and socialization skills were assessed. The multivariate logistic regression analysis was used to identify factors associated with DDs. The Adjusted Odds Ratio (AOR) with a 95% Confidence Interval was estimated to indicate the strength of association. A p-value of <0.05 was used to declare statistical significance.

**Results:**

Out of the 20324 surveyed school-aged children, 7.4% were found to have at least one delay. Communication deficits were the most common (6.4%) followed by delay in daily living skills (2.0%). The final model of logistic regression had a good fit for seven variables out of the sociodemographic, epidemiological characteristics, maternal and perinatal problems that were associated with a higher likelihood of at least one DD: Children suffering from any convulsions (AOR = 4.32; 95% CI: 3.18–5.88), male gender (AOR = 1.86; 95% CI: 1.65–2.09), birth weight less than 2.5 kg (AOR = 1.77; 95% CI: 1.40–2.24), history of maternal health problem during pregnancy (AOR = 1.64; 95% CI:1.34–2.01), children staying in an incubator for more than two days (AOR = 1.57, 95% CI: 1.29–1.91), having less educated fathers (AOR = 1.55, 95% CI: 1.24–1.95) and belonging to the middle social class (AOR = 1.40, 95% CI: 1.24–1.58).

**Conclusion:**

The identified types and determinants for each DD are allowing for the implementation of tailored programs for school children’s life skills promotion for achieving the most sustainable effects on children’s biological and psychological health and well-being.

## Background

A developmental delayappears when the child fails to achieve developmental goals as compared to peers of the same chronological age [1]. Many school children struggle with distinct types of developmental delays such as cognitive delay, motor delay, language delay, and social-emotional delay. These delays can impact their participation in learning and lead to inconsistent behaviors with the school or program expectations [2]. Cognitive delays may affect a child’s intellectual functioning, interfering with awareness and causing learning difficulties in addition to difficulty in communicating and playing with others [3]. Motor delay in school children manifests in having coordination problems that are serious enough to interfere with academic performance and social integrity [4]. Unobserved and unmanaged delayed language development can have a significant serious outcome affecting educational, social, and psychological progress [5]. Children with a socio-emotional delay may manage information or respond to their environment differently than children of the same age. With all these significant implications, it is mandatory to know the prevalence rates of developmental delays which is varied considerably between studies. The variations were mainly due to discrepancies in developmental levels between countries, differences in research methodology, and variations in definitions of developmental delay used in each study [6]. At the same time, the causes of developmental delay can be difficult to identify. However, many genetic and environmental factors can interfere with developmental trajectory and contribute to delays [7]. The existing evidence indicates that in developing countries, at least 20%–25% of the children are subjected to multiple risk factors affecting their development [8] including: sociodemographic factors, child and maternal physical health, and maternal mental health [9]. Identifying risk and protective factors associated with developmental delay, along with early intervention are necessary measures to help delayed children to achieve their developmental potential.

LSE programs are thought to be a solution for the delays due to the influential effect in improving young children’s capacity to develop and learn. LSE intends to empower students to deal efficiently with the challenges of everyday life by enhancing self-regulation, creating informed decisions, and developing supportive social relations [10]. Evaluation studies of LSE programs, mostly revealed inconsistent results, with significant positive, as well as zero outcomes [11]. This may be due to improper preparation and lack of needs assessment of the target groups before the development and implementation of the program. Egypt has started the LSE program with the early grades (preschool and grade 1) in 2017–2019 as a part of education transformation, the new curriculum is being progressively rolled out up to upper secondary education [12].

Unfortunately, the implementation of LSE in Egypt was lacking the full picture of the developmental delay situation as a determinant for the contents of the skills to be promoted. Screening of DDs among school-age children could be a driving factor in limiting the prevalence of developmental disabilities through the application of the appropriate life skills development programs. Our current study focused on providing the baseline situation of the importantly linked domains in enhancing the implementation of life skills programs. The current study aimed at identifying the prevalence and risk factors of motor, daily living skills, communication, and socialization delays at a national level. The results will help promote related life skills for coping with daily life problems, communication, social interaction, and problem-solving skills.

## Methods

### 1) Study design and setting

A Cross sectional national community-based study was conducted in 8 Governorates representing all geographic regions of Egypt according to their population density. The whole study was conducted over 24 months starting from December 2017 till December 2019.

### 2) Target group

The study targeted parents or caregivers of all children aged 6 years up to 12 years at the visited houses. Children who experienced normal milestones for their ages (6–12 years), as well as any child who met the definition of developmental delay, were included in the current study. A DD is defined as a delay in reaching milestones in one or more of the areas of development (gross and fine motor, speech-language, social-emotional, and cognitive skills) in an expected way for a child’s age [13]. Meanwhile, children with known developmental disabilities not delays were excluded. A developmental disability was defined as impairment of a body structure or function which occurs during developmental period accompanied with limitation of activities and restriction of participation [14]. The excluded children were those with diagnosed genetic disorders (e.g., Turner syndrome, Down syndrome, or Fragile-X syndrome) as assessed by a clinical genetics team from the National Research Centre of Egypt. The validated WHO ten-question screening tool with their probes was explained to the household for detecting any disability that is affecting hearing, vision, movement, learning, thinking, or social relationships [15, 16]. Children with detected disabilities were also excluded from the study.

Although these disabilities were beyond the scope of the current study, the researchers ensured that these children were enrolled in rehabilitation programs by the Ministry of Health.

### 3) Sampling frame and cluster preparation

**Fig 1:**
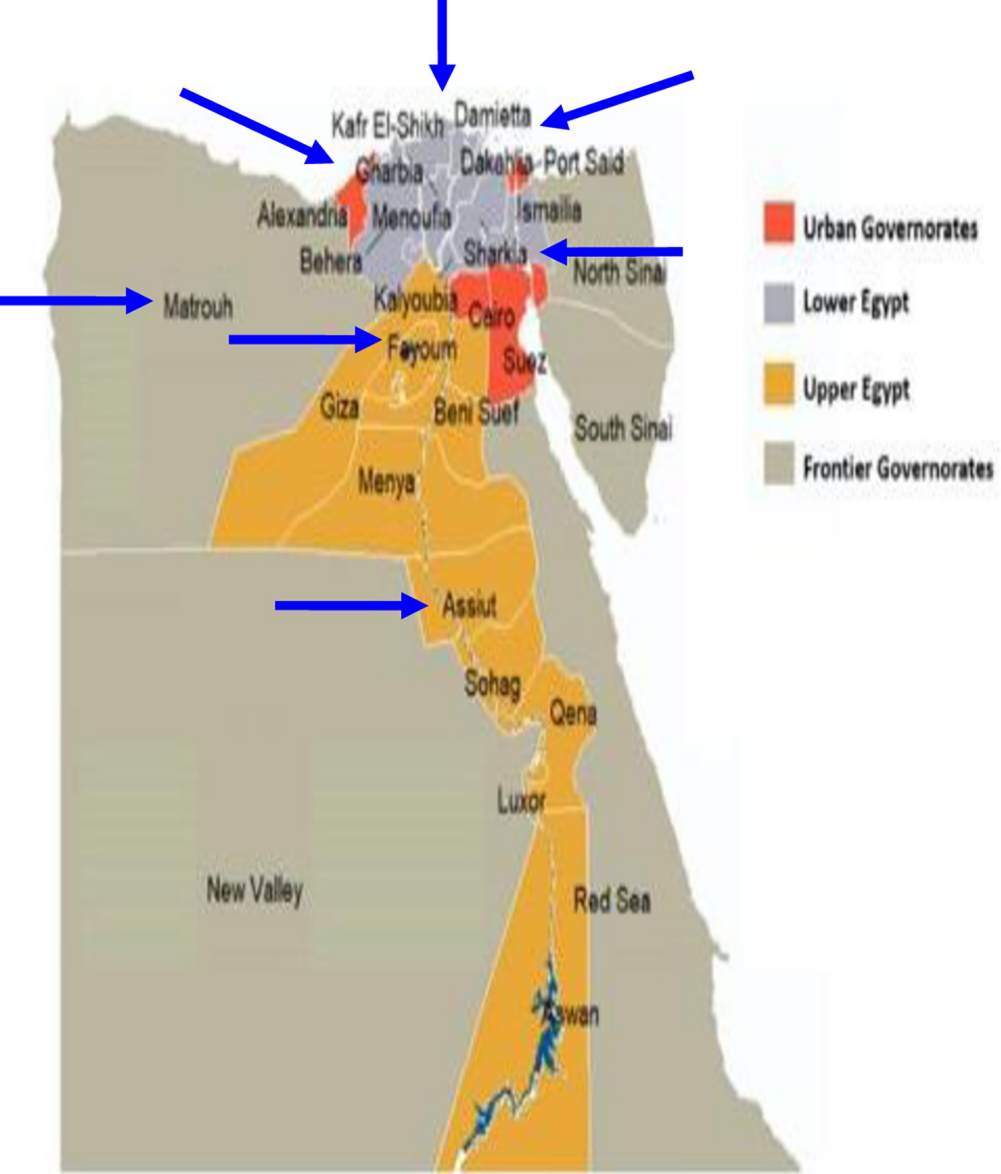
Map of the 27 Egyptian’s governorates distributed within the four geographic regions (adapted using data from the Humanitarian Data Exchange under the CC BY–IGO license [18].

A multistage cluster random sampling technique was used with three sampling frames as three stages; **The first sampling frame** used was the comprehensive list of the 27 governorates of Egypt, according to the enumeration census from the Central Agency for Public Mobilization and Statistics (CAPMAS) [17] within each of the four main geographic administrative regions of Egypt as shown in Fig 1.

**In the first stage**, 8 clusters of governorates were randomly selected. The selection of the governorates was according to the number and percent distribution of the population according to governorates in 2017 census which revealed that population percentage in urban governorates reached 17.1% of the total population, 43% in Lower Egypt versus 38% in Upper Egypt, while Frontier governorates only represented 1.7% of the total population in the same year. Accordingly, the following governorates were selected: One urban (Cairo), 3 Upper Egypt (Fayoum, Assuit, and Aswan), 3 Lower Egypt (Damietta, Dakahlia, and Gharbia) and one border -Frontier- (Marsa Matrouh).

Egyptian governorates are further subdivided into urban cities (kism) and rural local village unit (markaz). Egypt contains 177 cities and 162 local village units. **In the second stage**, a comprehensive list of the cities and local village units of each of the randomly chosen 8 governorates of Egypt was obtained. Using the UNFPA 2003 human development index that is based on the socio-economic status as a wealth index [19–22], three social categories; namely low, medium and high social class were identified. Each city and local village unit was assigned to a social category. Three cities and three local village units were randomly selected from each category.

**In the third stage,** all Shiakha and villages of each of the randomly selected Kism and Markaz were listed as clusters with random selection of one Shiakha and one village per social category of each of the chosen governorate. The study finally included 45-blocks of Shiaka and villages (within 24 Kism in urban areas and 21 markaz in rural areas respectively) to ensure heterogeneity of the data collected (S1 Table). In this stage, households in the selected city and villages blocks were screened.

The sample was allocated to be proportional to the size of large governorates. While governorates with relatively small populations were assigned to arbitrary sample sizes by adjusting weights during the analysis of the data.

### 4) Sample size

The sample size calculation is based on the estimated prevalence of 1%- 10% of the pediatric population with developmental delays [23]. The least prevalence was taken into consideration to ensure the largest sample size at a level of accuracy set at 0.001 (margin of error) with a width equal to 0.01 when the sample proportion is 0.12, confidence limit 97%. The large sample size will potentiate the reliability of results of the questionnaire used for detecting DD. Accordingly, targeting 20,091 children is expected to ensure the provision of estimates of DDs domains [24, 25]. The large representative sample in the screening phase aims at ensuring data accuracy. Due to the extended nature of families in some of the targeted houses (more than one family living in the same house) especially in rural areas, children who fulfilled the inclusion and exclusion criteria for this study for all caregivers within the visited houses were included in the survey. Children enrolled in the study were 20324. The following formula was used for estimating the sample size: N = z2 [P (1-P)/ D2], where Z = 2.58 and P = 0.5, D = 0.009.

### 5) Study instruments

The instrument utilized in the current study was a formulated questionnaire, reviewed by the researchers of the National Research Centre (NRC) and introduced by the well-trained surveyor to the parents, or guardians through a face-to-face interview. The data collected from this questionnaire were categorized into three components:

**The first component** delineated the sociodemographic and epidemiologic characteristics of the pupil and his parents, including the age of the pupil, gender, residence; whether urban or rural, locality, maternal age, and parental education and occupation, according to El-Gilany et al., 2012. Education levels of mothers or fathers were stratified into three categories: The first category included illiterate, read and write, primary, and preparatory, the second category included secondary (general & technical of 3 or 5 years) and Intermediate (2years) institutes, and the third category included university graduate and postgraduate degree. Maternal or paternal occupational status was classified into 2 categories; either non-employed including (non-working/house wife, and unskilled manual worker), and employed individual including; (skilled manual worker/farmer, trades/business, semi-professional/clerk, and professional) [26].

**The second component** included questions about the medical history of the assessed children and their mothers during the prenatal, natal, and post-natal time. This questionnaire comprised: mother’s age at giving birth which included 3 categories (> 18 years, 18–35 years, and > 35 years). The first age category was added because 17% of girls in Egypt are married before their 18th birthday [27]. A history of preterm delivery, low birth weight, twins, neonatal jaundice, cyanosis, convulsions or admission to Neonatal Intensive Care Unit for more than two days was recorded. Also maternal history of complications during pregnancy as gestational diabetes, hypertension, iron deficiency anemia, anxiety, depression or infection [28], or a history of difficult labor which refers to prolongation in the duration of labor, especially in the first stage of labor was included [29].

**The third component** intended for assessment of the five developmental domains (Gross motor, fine motor, daily living skills, communication, and social domain) through questions adapted from a reliable and diagnostic test; The Vineland Adaptive Behavior Scales (VABS) [30].

The Vineland Adaptive Behavior Scales (VABS) is a standardized assessment tool, that has been standardized for factors including gender, race, age, and parental education. It utilizes parent- interview to measure adaptive behavior and identify children with developmental delay in the domains of communication, daily living skills, social skills, and motor skills. VABS also includes a Maladaptive Behavior domain (with no subdomains), the administration of which is optional. This domain was not assessed in the current survey.

In the Communication Domain, subdomains of receptive, expressive, and written language are measured. The Daily Living Skills Domain includes subdomains of personal, domestic and community skills. The Socialization Domain addresses interpersonal relationships, play and leisure time, and coping skills subdomains. The Motor Skills domain measures both gross and fine motor skill subdomains till the age of five years.

Reliability estimates included interrater and inter-interviewer were from the low 0.70’s to high 0.80’s. Split-half reliability for the Adaptive Behavior Composite ranges from 0.93 to 0.97, while subdomains were in the 0.80s and 0.90s. Test–retest reliabilities ran mostly from the 0.80s to0 .90s. Investigation for validity based on the relationship between the scale and other scales that measure the same construct revealed a correlation coefficient at 0.7 [30, 31]. The Arabic version of VABS (VABSA) [32] was used in this study. It has been reported to have robust normative data, demonstrate high reliability and validity, and yield useful diagnostic information [33]. It can be used in any Arabic speaking culture to assess adaptive and maladaptive behaviors, identify young children with disabilities, and to provide guidelines in offering services appropriate to these children [34].

In parental interview of VABS or VABSA, each item is scored on three levels; if the child usually performs the activity the score is 2, if he sometimes or partially performs the activity, the score is 1, if he never performs this activity the score is 0. VABS usually takes about 20–60 minutes to be completed.

In this study, some modifications were applied to VABSA [32] to cope with the time limit factor (Maximum 20 minutes for each child), the screening nature of the study, and the very large sample size (20324 children aged 6 to 12). The items of subdomains were written on 7 cards according to the child’s chronological age. Each card represented 1- year interval. Starting from the age of 6 and the last card ending at the age of 12. Each card included skills achieved at that age involving items of the main 4 domains (communication, daily living skills, social skills, and motor skills). Items were put in the form of Yes or No questions. If the student can usually perform the activity described, the answer will be Yes. If he can partially perform or he cannot perform the activity, the answer will be No. Partial performance of the activity (sometimes needs help or sometimes needs to be reminded) is considered a failure to avoid missing cases. For example: if the child was requested to read 10 words: if read 10 words clearly, the answer will be Yes, but if sometimes the child could not read some of these words the answer will be No. A delay in a specific domain is considered if the score of that domain is 2 SD below the mean (<70). Some questions relating to motor functioning in school-age children were integrated in the questionnaire according to Matheis and Estabillo, 2018. These questions aim to delineate any impairment in gross or fine motor development [35].

Recognized children with developmental delays were referred to physicians in the health care centers of the Ministry of Health and Population (MOHP) to ascertain the results of the screening test. The affirmative delay was proved in 85% of the referred children.

### 6) Survey implementation

Before the survey implementation, a preparation phase was done to ensure quality control and contact local authorities. Condensed training sessions about how to conduct the questionnaire in a standardized way were done for 64 social workers (average 6/governorate). The survey was conducted under the supervision of a collaborative team from the Cairo Demographic Center (CDC) with professional team members from the National Research Centre of Egypt (NRC). A pilot study was performed on 80 participants (10/governorate) to ensure the validity of the questionnaire items through revising and modifying difficulty- understood items or language and then re-introduced them.

### 7) Statistical analysis and data processing

Data was entered into the Household Registration System (HRS) version 2.1. Data were analyzed using Statistical Package for the Social Sciences (SPSS) version 22.0 software (IBM SPSS Statistics for Windows, Version 22.0. Armonk, NY: IBM Corp) [36]. Descriptive statistics were first used to calculate the frequency distribution of the sociodemographic, epidemiological, maternal and perinatal factors that were related to DDs. All data were represented by both column and row percentages. The denominator was fixed for all calculations which is the total number of children aged 6–12 years enrolled in the study (n = 20324). The denominator for the calculation of the proportion of the studied sociodemographic variables that were related to children’s parents was also the total number of children enrolled in the study (n = 20324). We did not exclude any missed values from the denominator but they were excluded from the nominator during proportion calculation. This was the way used for handling the missing values for mother’s age at giving birth, mothers education, and fathers education to provide proportion of the real situation.

Comparisons between related variables were done using odds ratios (OR), and 95% confidence intervals (CI) were calculated in comparison between DDs and children without delays as two steps. Factors that were found to be statistically significant in the univariate logistic regression analysis were subjected to multivariate logistic regression (Enter Wald) for adjusting and controlling the effect of confounding variables to determine the predictors for the at least DD and domain‑dependent. Results were presented in terms of crude odds ratio (COR) and adjusted odds ratio (AOR) in a univariate and multivariate analysis respectively. Variables with p-values of <0.2 during the bivariable analysis were fitted to the multivariable logistic regression analysis. A significant association is considered if the 95% CI does not include the value 1.0. Finally, a cutoff *p*-value of less than 0.05 is used to declare statistical significance.

### 8) Ethical approval and consent to participate

The study was conducted after getting the approval of the Medical Research Ethics Committee of the National Research Centre with an ethical approval number of 17034. A written informed consent was taken from the parents/ guardians of all children enrolled in the study. For participants who were unable to write, a right thumb print was used as a signature. The study was fully voluntary, and data was collected in a confidential manner. Data was de-identified and stored in a safe location. The conduct of the study complied with the International Ethical Guidelines for Biomedical Research Involving Human Subjects [37]. The information disclosure for “Making sure parents/ guardians understand” was guaranteed according to the recommendations of the Egyptian patients and guardians’ perception that clinical informed consent is preferred purpose for Informed Consent practices [38].

## Results

**Fig 2:**
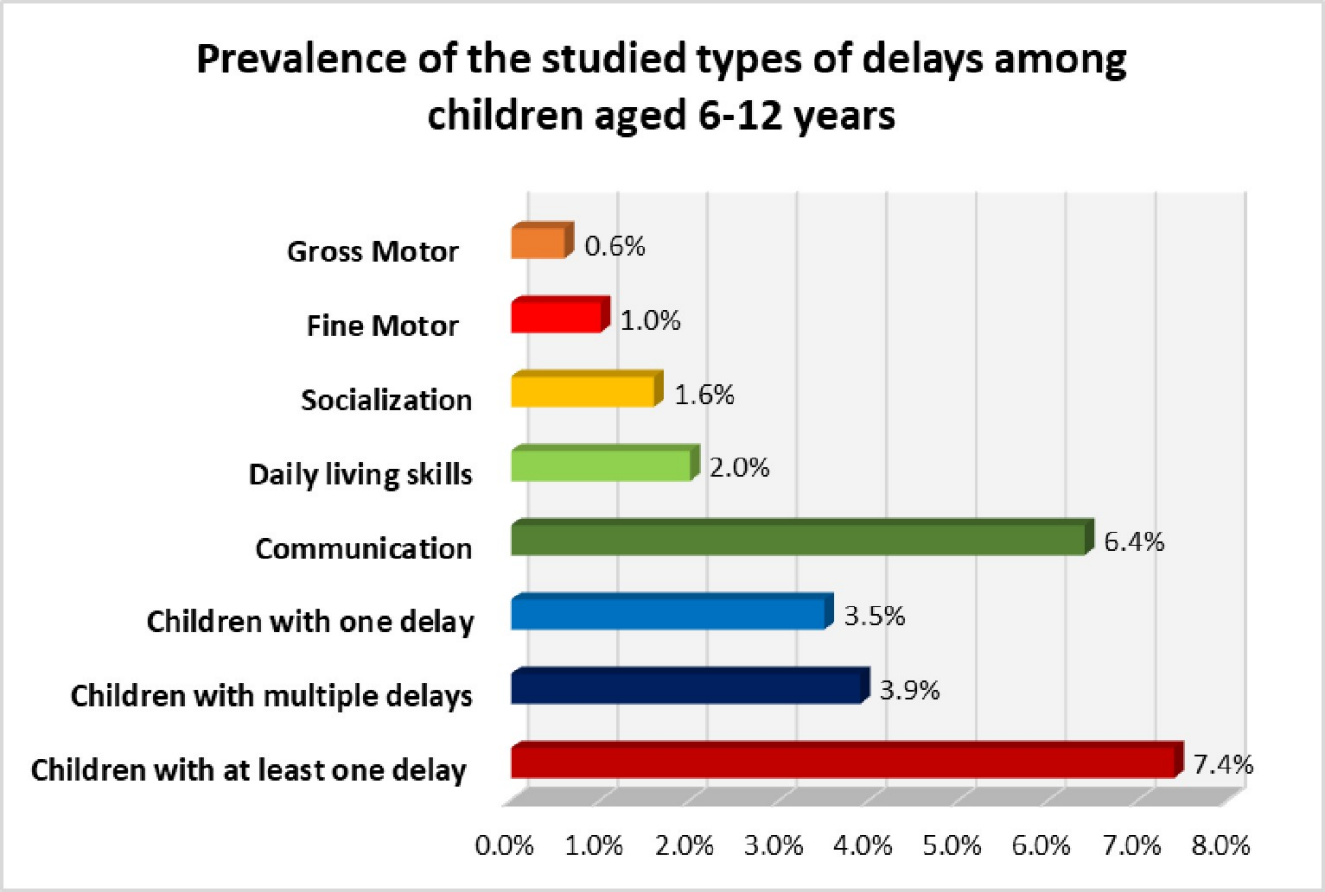
Prevalence of the studied types of delays among school children aged 6–12 (n = 20324).

The prevalence of Egyptian primary school children with at least one DDs was 7.4%, 3.5% with one delay, and 3.9% with multiple DDs. Overall maximum prevalence was seen in communication delay (6.4%) followed by daily living skills area (2.0%) followed by socialization deficits (1.6%). One percent of children had a fine motor delay while 0.6% had a gross motor delay which was the least prevalent delay Fig 2.

Table 1 showed the characteristics of the study population. A total of 20324 parents participated in the study. Children were slightly higher in rural communities than in urban ones. They were equally distributed between social classes. They were distributed among governorates proportional to their population size. The surveyed boys were slightly higher than girls (51% versus 49% respectively). Nearly 95.7% of them went to school. Most of the mothers were giving birth between the ages of 18 and 35 (86.5%). Regarding education levels, 15.8% of fathers versus 15% of mothers had completed their university degrees. Nearly half of mothers and fathers had high school or technical and above intermediate (45.2% and 41.9% respectively). Most of the mothers were unemployed (84.1%). Houses without fathers were 5.2% versus 0.9% without mothers. Among the perinatal problems, the presence of a history of neonatal jaundice was the most prevalent (23.9%) followed by a history of difficult labor (13.1%). Disabled mothers were only 1%.

**Table 1:** Characteristics of the study population.

Characteristics	Surveyed children (20324)	
	N	Column %
Locality		
Urban	9715	47.8
Rural	10609	52.2
Social class		
Low	6662	32.8
Middle	6794	33.4
High	6868	33.8
Geographical Distribution		
Cities	3655	18.0
Lower Egypt	7971	39.2
Upper Egypt	6639	32.7
Frontier	2059	10.1
Sex		
Boys	10361	51.0
Girls	9963	49.0
Mother age at giving birth		
< 18 years*	1085	5.3
18 to < 35 years	17576	86.5
≥35 years	1468	7.2
Mothers Education		
Illiterate/ Read & write/ Primary/ Prep	7896	38.9
High School & technical/ above intermediate	9177	45.2
University or higher	3052	15.0
Fathers Education		
Illiterate/ Read & write/ Primary/ Prep	7537	37.1
High School & technical/ above intermediate	8523	41.9
University or higher	3213	15.8
Mothers´ work		
work (paid-unpaid-her own-employer)	3038	15.9
unemployed / does not look for work	17093	84.1
Presence of mothers or fathers		
No father in the household	1051	5.2
No mother in the household	189	0.9
Perinatal problems		
Children with history of at least one perinatal problem	5660	27.8
Premature children (< 37 weeks gestation)	186	0.9
Low birth weight (< 2500 mg)	771	3.8
Children suffer from jaundice after birth	4848	23.9
Children suffer from cyanosis after birth	233	1.5
Children suffer from any convulsions	320	1.6
Children kept in an incubator for more than two days	1308	6.4
Child suffer from meningitis	188	0.93
Twins	799	3.9
Maternal problems		
Mothers have any health problem during pregnancy**	1270	6.2
Difficult labor***	2657	13.1

*This category is added because 17% of girls in Egypt are married before the age of 18 [27]

**Mothers having complication during pregnancy as gestational diabetes, hypertension, iron deficiency anemia, anxiety, depression or infection [28]

***Difficult labor refers to prolongation in the duration of labor, especially in the first stage of labor. It can be a contributor to maternal mortality and morbidity if unrecognized or untreated [29]^.^

Table 2 shows the odds of having DDs. Concerning the sociodemographic factors, the odds of the presence of at least one delay was nearly one and a half significantly higher among children living in urban communities (8.8%) than those living in rural areas (COR R = 1.47, 95% CI: 1.33–1.64). Children belonging to the middle social class (9.1%) were 1.4 times more likely to have at least one delay than those belonging to low or high social class (COR = 1.41, 95% CI: 1.24–1.60 & COR = 1.43, 95% CI: 1.26–1.63 respectively).

**Table 2:** Odds of having developmental delay among school children aged 6–12 years according to the sociodemographic characteristics.

Socio-demographic parameters N = 20324	Gross Motor (GM) N (%)	Fine Motor (FM) N (%)	Daily living skills N (%)	Communication N (%)	Socialization N (%)	Children with at least one delay N (%)
**Locality**						
**Urban (n = 9715)**	59 (0.6)	89 (0.9)	209 (2.2)	729 (7.5)	186 (1.9)	856 (8.8)
**Rural (n = 10609)**	72 (0.7)	113 (1.1)	195 (1.80)	563 (5.3)	139 (1.3)	653 (6.2)
COR **(C) Urban vs. rural**	0.89	0.86	1.17	1.45	1.47	1.47
	0.63–1.26	0.65–1.13	0.96–1.43	1.29–1.62*	1.18–1.84*	1.33–1.64*
**Social class**						
**Low (n = 6662)**	41 (0.6)	71 (1.1)	111 (1.7)	379 (5.7)	88 (1.3)	443 (6.6)
**Middle (n = 6794)**	42 (0.6)	62 (0.9)	164 (2.4)	509 (7.5)	138 (2.0)	618 (9.1)
**High (n = 6868)**	48 (0.7)	69 (1)	129 (1.9)	404 (5.9)	99 (1.4)	448 (6.5)
COR **Middle vs. low**	0.86	0.85	1.34	1.55	1.90	1.41
	0.61–1.20	0.60–1.20	1.17–1.54*	1.18–2.03*	1.57–2.30*	1.24–1.60*
COR **Middle vs. high**	0.91	0.91	1.30	1.42	1.69	1.43
	0.64–1.28	0.64–1.28	1.13–1.48*	1.09–1.84*	1.40–2.03*	1.26–1.63*
COR **low vs. high**	1.06	0.89	0.96	0.92	0.88	1.02
	0.76–1.48	0.69–1.14	0.84–1.12	0.69–1.22	0.72–1.10	0.89–1.17
**Geographical Distribution**						
**Cities (n = 3264)**	91(2.8)	23 (0.6)	40 (1.1)	102 (2.8)	390 (10.7)	467 (12.8)
**Lower Egypt (n = 7921**	112 (1.4)	64 (0.8)	101(1.3)	152 (1.9)	489 (6.1)	562 (7.1)
**Upper Egypt (n = 7705)**	168 (2.2)	34 (0.5)	48 (0.7)	116 (1.7)	326 (4.9)	369 (5.6)
**Frontier (n = 2426)**	41 (1.7)	10 (0.5)	13 (0.6)	34 (1.7)	87 (4.2)	111 (5.4)
COR **lower vs cities**	1.28	1.16	0.68	0.55	0.53	0.52
	0.79–1.06	0.80–1.68	0.53–0.87*	0.48–0.63*	0.40–0.70*	0.46–0.59*
COR **upper vs cities**	0.81	0.66	0.62	0.43	0.57	0.40
	0.48–1.38	0.43–1.00	0.47–0.81*	0.37–0.50*	0.43–0.76*	0.35–0.46*
COR **Frontiers vs cities**	0.77	0.57	0.59	0.37	0.71	0.39
	0.37–1.62	0.31–1.08	0.40–0.87*	0.29–0.47*	0.48–1.04	0.31–0.48*
COR **lower vs. Frontiers**	1.66	2.02	1.16	1.48	0.75	1.33
	0.85–3.24	1.13–3.61*	0.80–1.69	1.17–1.87*	0.51–1.10	1.08–1.64*
COR **upper vs. Frontiers**	1.06	1.15	1.06	1.17	0.81	1.03
	0.52–2.14	0.62–2.12	0.72–1.56	0.92–1.49	0.55–1.19	0.83–1.29

* = p–value significant at <0.05

Regarding all the studied developmental domains, belonging to the middle social class carried a significant one and a half higher odds than both the high and low social class for all domains except for a delay in the motor milestones. Living in urban communities carried a significantly higher odds of delay for communication and socialization domains (COR = 1.45,95% CI:1.29–1.62 & COR = 1.47, 95% CI:1.18–1.84 respectively).

Generally speaking, living in cities is significantly associated with at least one DD (12.8%). Living in lower or upper Egypt significantly decreased the odds of delay than living in cities for the three domains in a range of 35% to 55% except for detecting delay in both the gross and fine motor domains. Living in frontiers significantly decreased the odds of delay than living in cities for daily living skills by 40% (COR = 0.59, 95% CI: 0.40–0.87) and for communication by 62% (COR = 0.37, 95% CI: 0.29–0.47).

Meanwhile, living in lower Egypt significantly increased the odds of fine motor delay by twice and the odds of communication delay by one and a half if compared with living in frontiers (COR = 2.02, 95% CI: 1.13–3.61 & COR = 1.48, 95% CI: 1.17–1.87 respectively).

The proportion of epidemiological factors that were significantly associated with at least one DD out of the total surveyed is shown in Table 3 and was found to be: being male child (9.4%), age of mothers at giving birth above 35 years (8.7%), living without mothers (11.8%) and/or fathers (11.0%) in homes, children with mothers or fathers who were illiterate or below high school education (8.5% & 8.8% respectively).

**Table 3:** Odds of having developmental delay among school children aged 6–12 years according to the Epidemiological characteristics.

Epidemiological characteristics n = 20324	Gross Motor N (%)	Fine Motor N (%)	Daily living skills N (%)	Communication N (%)	Socialization N (%)	Children with at least one delay N (%)
**Sex of the child**						
**Male (n =** 10361)	81 (0.8)	127 (1.2)	270 (2.6)	862 (8.3)	220 (2.1)	974 (9.4)
**Female (n =** 9963)	50 (0.5)	75 (0.8)	134 (1.3)	430 (4.3)	105 (1.1)	535 (5.4)
COR (CI) males/Females	1.56	1.64	1.96	2.01	2.04	1.83
	1.10–2.23*	1.23–2.18*	1.59–2.42*	1.79–2.27*	1.62–2.57*	1.64–2.04*
**Mother’s age at giving birth**						
**< 18 (**n = 91085)	5 (0.5)	5 (0.5)	13 (1.2)	50 (4.6)	16 (1.5)	58 (5.3)
**18 to < 35** (n = 17576)	107 (0.6)	173 (1)	343 (2)	1107 (6.3)	271 (1.5)	1299 (7.4)
**≥35 (**n = 1468)	169 (1.1)	20 (1.4)	39 (2.7)	115 (7.8)	29 (2)	128 (8.7)
COR ≥ 35 vs. <18	2.38	2.98	2.25	1.76	1.35	1.69
	0.87–6.52	1.12–7.94**	1.20–4.24**	1.25–2.48**	0.73–2.49	1.23–2.33*
COR 18-<35 vs. <18	1.32	2.15	1.64	1.39	1.05	1.41
	0.54–3.25	0.88–5.24	0.94–2.87	1.04–1.86*	0.63–1.74	1.08–1.85*
COR 18-<35 vs. ≥ 35	0.56	0.72	0.73	0.79	0.78	0.84
	0.33–0.94*	0.45–1.15	0.52–1.02	0.65–0.97*	0.53–1.14	0.69–1.101
**No mother (n = 186)**	3 (1.6)	4 (2.2)	9 (4.8)	18 (9.7)	9 (4.8)	22 (11.8)
COR no mother vs. mother at home	2.56	2.21	2.54	1.59	3.19	1.68
	0.81–8.12	0.81–6.02	1.29–5.00*	0.97–2.59	1.62–6.29	1.08–2.64
**No father (n = 1050)**	10 (1)	14 (1.3)	31 (2.9)	93 (8.8)	35 (3.3)	116 (11)
COR no father vs. father at home	1.52	1.37	1. 54	1.46	2.26	1.59
	0.80–2.91	0.79–2.37	1.06–2.23*	1.17–1.83*	1.58–3.22*	1.30–1.95*
**Mothers Education**						
**1) Illiterate/ below high school (n = 7896)**	55 (0.7)	80 (1)	183 (2.3)	596 (7.5)	144 (1.8)	671 (8.5)
**2)High School (n = 9177)**	60(0.7)	102(1.1)	176 (1.9)	574 (6.3)	147 (1.6)	682 (7.4)
**3. University or higher (n = 3052)**	13(0.4)	16(0.5)	36 (1.2)	104 (3.4)	25 (0.8)	134 (4.4)
COR 2 vs. 3	1.54	2.13	1.64	1.89	1.97	1.75
	0.84–2.81	1.26–3.62*	1.14–2.35*	1.53–2.34*	1.29–3.02*	1.45–2.11*
COR 2 vs. 1	0.94	1.10	0.82	0.82	0.88	0.86
	0.65–1.35	0.82–1.47	0.67–1.02	0.73–0.92*	0.70–1.11	0.77–0.97*
COR 3 vs. 1	0.61	0.52	0.50	0.43	0.45	0.49
	0.33–1.12	0.30–0.88*	0.35–0.72*	0.35–0.53*	0.29–0.68*	0.41–0.60*
**Fathers Education**						
**1) Illiterate/ below high school (n = 7537)**	55(0.7)	84(1.1)	185(2.5)	591(7.8)	137(1.8)	660(8.8)
**2)High School (n = 8523)**	54(0.6)	82(1)	147(1.7)	496(5.8)	129(1.5)	590(6.9)
**3. University or higher (n = 3213)**	12(0.4)	22(0.7)	41(1.3)	112(3.5)	24(0.7)	143(4.5)
COR 2 vs. 3	1.70	1.41	1.36	1.71	2.04	1.60
	0.91–3.18	0.88–2.26	0.96–1.92	1.39–2.11*	1.32–3.16*	1.32–1.93*
COR 2 vs. 1	0.87	0.86	0.70	0.73	0.83	0.78
	0.60–1.26	0.64–1.17	0.56–0.87*	0.64-.82*	0.65–1.06	0.69–0.87*
COR 3 vs. 1	0.51	0.61	0.51	0.42	0.41	0.49
	0.27-.95*	0.38–0.98*	0.37–0.72*	0.35–0.52*	0.26–0.63*	0.40–0.58*
**Mothers´ work**						
**1. Employed (n = 3038)**	18 (0.6)	34 (1.1)	58 (1.9)	162 (5.3)	47 (1.5)	195 (6.4)
**2.unemployed (n = 17093)**	110 (0.6)	164 (1)	337 (2)	1112 (6.5)	269 (1.6)	1292 (7.6)
COR 2 vs. 1	1.09	0.86	1.03	1.24	1.02	1.19
	0.66–1.79	0.59–1.24	0.78–1.37	1.04–1.46*	0.75–1.39	1.02–1.39*

* = p–value significant at <0.05 * = sig

** = Highly sig.

Concerning the odds of the epidemiological factors (Table 3), boys significantly carried about twice the odds to have a delay in the five studied domains than girls. The lowest odds were observed in the gross motor domain (COR = 1.56, 95% CI: 1.10–2.23), the highest odds were presented in socialization domain (COR = 2.04, 95% CI:1.62–2.57). The age of mothers at giving birth above 35 years significantly increased the odds of FMD (COR = 2.98, 95% CI: 1.12–7.94), daily living skills (COR = 2.25, 95% CI: 1.20–4.24), and communication delay (COR = 1.76, 95% CI: 1.25–2.48) than mothers giving birth below 18 years. Mothers giving birth at the age range 18 to <35 years significantly decreased the odds of having children with both GMD by 44% and communication deficits by 21% (COR = 0.56, 95% CI: 0.33–0.94 & OR = 0.79, 95% CI: 0.65–0.97 respectively) than mothers giving birth at age > 35 years. Meanwhile, living without mothers and/or fathers in homes increased the odds of having at least one delay by nearly one and half times (COR = 1.68, 95% CI: 1.08–2.64 and COR = 1.59, 95% CI: 1.30–195 respectively). Whereas living without mothers carried almost three times the odds of delay in the socialization domain (COR = 3.19, 95% CI: 1.62–6.29) and in the daily living skills (COR = 2.54, 95% CI: 1.29–5.00). Meanwhile, living without a father or a mother carried almost the same odds of delay with the communication domain (COR = 1.46, 95% CI: 1.17–1.83 and COR = 1.59, 95% CI: 0.97–2.59 respectively).

Children with mothers or fathers who had higher education were less likely to have any type of delays, especially those who had a college or greater education level with fewer odds of a range from 23% to 50% than lower grades of education. Mothers’ work did not affect the odds of having any delay.

The odds of having developmental delay for all the studied developmental domains according to all the studied medical perinatal history problems were shown in Table 4. All the studied medical histories for both mothers and children were risk factors for all domains except for GMD concerning the risk for mothers with a history of any health problem during pregnancy or children suffering from jaundice after birth.

**Table 4:** Odds of having developmental delay according to medical perinatal history problems and postnatal child problems.

Type of the risk §	Total children Surveyed N = 20324				
	Gross Motor n = 131	Fine Motor n = 202	Daily living skills n = 404	Communication n = 1292	Socialization n = 325
	N (%)	N (%)	N (%)	N (%)	N (%)
Mothers having any health problem during pregnancy (n = 1270)	17 (1.3)	35 (2.8)	57 (4.5)	172 (13.5)	37 (2.9)
COR (CI)	2.25	3.21	2.53	2.51	2.00
	0.35–3.76	2.22–4.64**	1.90–3.37**	2.11–2.98**	1.38–2.77**
Difficult labor (n = 2657)	35 (1.3)	59 (2.2)	100 (3.8)	260 (9.8)	68 (2.6)
COR (CI)	2.44	2.78	2.23	1.75	1.78
	1.66–3.61**	2.05–3.78**	1.78–2.81**	1.52–2.02*	1.36–2.33*
Premature child (< 37 weeks gestation) n = 186	10 (5.4)	13 (7.0)	21 (11.3)	34 (18.3)	16 (8.6)
COR (CI)	9.51	8.03	6.65	3.40	6.11
	4.91–18.42**	4.49–14.4**	4.17–10.6**	2.33–4.96**	3.62–10.33**
Low birth weight (< 2500 mg at birth) n = 771	10 (1.3)	22 (2.9)	58 (7.5)	127 (16.5)	39 (5.1)
COR (CI)	2.11	3.16	4.52	3.11	3.59
	1.10–4.04*	2.02–4.95**	3.39–6.02**	2.55–3.80**	2.55–5.06**
Child suffer from jaundice after birth (n = 4848)	40 (0.83)	71 (1.5)	136 (2.8)	439 (9.1)	108 (2.2)
COR (CI)	1.41	1.74	1.64	1.71	1.60
	0.97–2.04	1.30–2.33*	1.33–2.02*	1.51–1.92*	1.27–2.02*
Child suffer from cyanosis after birth (n = 233)	15 (6.4)	18 (7.7)	36 (15.5)	63 (27.0)	28 (12.0)
COR (CI)	11.85	9.06	9.79	5.69	9.10
	6.81–20.62**	5.48–14.97**	6.77–14.2**	4.24–7.64**	6.03–13.73**
Child suffer from any convulsions (320)	20 (6.3)	24 (7.5)	49 (15.3)	101 (31.6)	45 (14.1)
COR (CI)	11.95	9.03	10.01	7.29	11.53
	7.32–19.49**	5.81–14.04**	7.25–13.8**	5.71–9.29**	8.23–16.14**
Child kept in an incubator for more than two days (1308)	27 (2.1)	39 (3.0)	71 (5.4)	204 (15.6)	68 (5.2)
COR (CI)	3.83	3.56	3.22	3.05	4.00
	2.50–5.88**	2.50–5.06**	2.48–4.19**	2.59–3.58**	3.05–5.26**
Child suffer from meningitis (n = 188)	6 (3.2)	10 (5.3)	23 (12.2)	34 (18.1)	17 (9.0)
COR (CI)	5.28	5.84	7.23	3.31	6.40
	2.30–12.13**	3.04–11.21**	4.62–11.3**	2.28–4.82**	3.84–10.67**

§ = row %

* = sig.

** = Highly sig.

The odds of DDs according to domain type is largely affected by the type of the medical perinatal history. Mothers having a history of any health problem during pregnancy or history of difficult labor carried almost three times the odds of FMD than children born to healthy mothers (COR = 3.21, 95% CI: 2.22–4.64 & COR = 2.78, 95% CI: 2.05–3.78 respectively). Whereas most neonatal health problems carried higher odds of GMD than being a healthy child. Children with a history of cyanosis or convulsions carried 11 times higher odds of GMD than healthy children (COR = 11.85, 95% CI: 6.81–20.62 & COR = 11.95, 95% CI: 7.32–19.49 respectively). The odds of daily living skills delay were found with a history of LBW children and those with a history of meningitis after birth (COR = 4.52, 95% CI: 3.39–6.02 & COR = 7.23, 95% CI: 4.62–11.3 respectively). History of preterm delivery carried more than eight odds of both the GMD and FMD than those born at their full gestational age (COR = 9.51, 95% CI: 4.91–18.42 & COR = 8.03, 95% CI: 4.49–14.4 respectively). Children with a history of jaundice carried the highest odds of the delay in FM (COR = 1.74, 95% CI:1.3–2.33) followed by a communication delay (COR = 1.71, 95% CI:1.51–1.92). A child kept in an incubator for more than two days after birth carried more than 3 times higher odds of GMD (COR = 3.83, 95% CI:2.50–5.88) which is nearly the same as other types of delay.

Table 5 shows the data of the multivariate logistic regression model for predictors of both the DDs as well as each type of the studied developmental domains among children aged 6–12 years. Seventeen significant variables that were associated with the presence of at least one DD versus healthy children (as evident from the univariate analysis) were entered into a multivariate logistic regression model using the enter selection procedure to explore the predictors of DD domains. These variables included: eight sociodemographic/epidemiological, two maternal, and seven children risk factors. The final model had a good fit for seven variables increasing the association for at least one DD (three sociodemographic/epidemiological, two maternal, and two children risk factors). Risk factors in order were: Children suffering from any convulsions (AOR = 4.32; 95% CI: 3.18–5.88), being a boy child (AOR = 1.86; 95% CI: 1.65–2.09), children with birth weight less than 2.5 kg (AOR = 1.77; 95% CI: 1.40–2.24), mothers having any health problem during pregnancy (AOR = 1.64; 95% CI:1.34–2.01), children kept in an incubator for more than two days (AOR = 1.57, 95% CI: 1.29–1.91) and less educated fathers (AOR = 1.55, 95% CI: 1.24–1.95).

**Table 5:** Multivariate Logistic regression model for prediction of delays versus children without delay: Gross motor delay (GM), fine motor delay (FM), Self–help & adaptive behavior delay (SA), Language /Cognitive delay (LC), Social–emotional delay (SE) and Children with at least one delay.

*Parameters*	gross motor (GM)			fine motor			Daily living skills			Communication				Socialization			Children with at least one delay		
* *	Wald	AOR	CI	Wald	AOR	CI	Wald	AOR	CI	Wald		AOR	CI	Wald	AOR	CI	Wald	AOR	CI
**Sex**	5.5	1.6	1.6–2.3*	10.6	1.7	1.2–2.3*	33.9	1.9	1.5–2.4*	118.		2.2	1.7–2.3*	35.1	2.2	1.7–2.8*	107.3	1.86	1.65–2.09*
**Locality: urban is the base**																			
**urban**	0.11	0.92	0.5–1.5	2.3	0.7	0.50–1.1	0.05	1.0	0.74–1.3	4.9		1.2	1.0–1.4*	1.05	1.2	0.9–1.6	2.63	1.13	0.98–1.31
**Social class: Middle is the base**																			
**Middle**	0.14	0.92	0.6–1.40	0.87	0.85	0.61–1.2	8.6	1.4	1.1–1.7*	16.4		1.3	1.1–1.5*	5.1	1.3	1.0–1.7*	30.7	1.40	1.24–1.58*
**Geographical distribution: (Lower, Upper and Frontiers) are the base**																			
Lower	0.10	1.1	0.60–2.0	0.74	0.80	0.50–1.3	4.4	0.69	0.49–0.98*	29.4		0.59	0.49–0.71*	6.9	0.60	0.41–0.88*	45.6	0.54	0.46–0.65*
**Upper**	1.5	0.65	0.3–1.3	7.0	0.50	0.30–0.82*	6.1	0.64	0.45–0.91*	65.8		0.44	0.36–0.53*	4.8	0.65	0.44–0.96*	93.3	0.40	0.33–0.48*
**Frontiers**	0.90	0.67	0.3–1.5	3.7	0.50	0.26–1.0	4.8	0.61	0.39–0.95*	61.6		0.35	0.2–0.46*	.80	0.82	0.53–1.27	62.7	0.39	0.31–0.49*
**Maternal education: University and above is the base**																			
**University and above**	2.4	0.53	0.24–1.2	12.00	0.29	0.14–0.58*	11.50	0.43	0.27–0.70*	37.8		0.42	0.32–0.55*	5.97	0.48	0.27–0.87*	35.2	0.46	0.36–0.60*
**Paternal education: Less education is the base**																			
**Less educated**	0.98	1.46	0.69–3.1	0.09	0.92	0.53–1.5	1.07	1.2	0.82–1.9	15.4		1.6	1.3–2.1*	4.9	1.8	1.1–3.0*	14.6	1.55	1.24–1.95*
**Maternal problems are the base**																			
**Health problem during pregnancy**	0.01	0.96	0.49–1.9	3.5	1.6	1.0–2.5*	0.56	1.15	0.80–1.7	15.6		1.5	1.2–1.9*	0.38	0.86	0.54–1.4	23.3	1.64	1.34–2.01*
**difficult labor**	2.9	1.6	0.94–2.5	6.1	1.6	1.1–2.4*	8.7	1.54	1.2–2.1*	7.18		1.3	1.07–1.5*	2.66	1.3	0.94–1.9	4.41	1.20	1.01–1.41*
**Perinatal problems are the base**																			
**Preterm delivery**	3.21	2.7	0.91–7.8	0.70	1.45	0.59–3.8	1.73	0.59	0.27–1.3		4.46	0.65	0.32–1.06	0.87	0.65	0.27–1.60	5.88	0.59	0.31–1.01
**LBW**	0.48	0.73	0.31–1.8	0.40	1.22	0.66–2.2	19.32	2.35	1.61–3.4*		20.3	1.8	1.38–2.26*	3.57	1.58	0.98–2.55	22.5	1.77	1.40–2.24*
**Suffering from jaundice after birth**	1.93	0.72	0.44–1.15	0.40	0.891	0.62–1.3	0.01	0.99	0.76–1.3		3.59	1.1	0.99–1.33	0.11	0.95	.71–1.28	3.50	1.14	0.99–1.30
**Suffering from Cyanosis after birth**	7.68	3.4	1.43–7.9*	4.33	2.3	1.1–4.8*	5.53	2.02	1.12–3.6*		3.57	1.5	0.99–2.29	1.42	1.52	.77–3.0	1.50	1.29	0.86–1.96
**Suffering from any convulsions**	22.18	5.35	2.7–10.7*	16.2	3.62	1.9–6.8*	32.19	3.95	2.5–6.4*		64.9	3.8	2.73–5.21*	65.5	6.99	4.36–11.1*	87.1	4.32	3.18–5.88*
**Kept in an incubator for more than two days**	7.23	2.23	1.2–4.0*	7.318	1.93	1.2–3.1*	2.45	1.34	0.93–1.9		22.7	1.7	1.34–2.03*	11.4	1.96	1.3–2.89*	19.7	1.57	1.29–1.91*
**Suffering from meningitis**	1.24	0.48	0.13–1.7	0.001	0.98	0.35–2.7	2.08	1.65	0.84–3.3		0.77	0.79	0.46–1.35	0.01	1.05	0.47–2.33	.04	0.95	0.58–1.56
**Constant**	0.384	1.9		14.4	20.7		15.5	0.14			87.17	0.06		95.47	0.003		67.97	0.103	

Four sociodemographic/epidemiological factors were found to be protective against the occurrence of at least one DD. These protective factors were: being resident in Frontiers, Lower or Upper Egypt which decrease the odds to have DDs by 61% 60% & 46% compared to living in cities (AOR = 0.39, 95% CI: 0.31–0.49, AOR = 0.40, 95% CI: 0.33–0.48 & AOR = 0.54, 95% CI: 0.46–0.65), as well as maternal education with a university degree or above degree which decreases the odds to have DDs by 54% compared to being with less education (AOR = 0.46, 95% CI: 0.36–0.60).

When considering each of the developmental domains, the odds of all delays were higher among male children than being females with a range of odds of delay that varied from one and a half for GM (AOR = 1.6, 95% CI:1.6–2.3) to more than twice for socialization delay (AOR = 2.2, 95% CI:1.7–2.8). The strongest predictors for any DDs domains were in order children with a neonatal history of convulsions or LBW or kept in an incubator for more than two days after birth and if mothers had a history of any health problems during pregnancy. Whereas the history of neonatal convulsions acts as risk predictors for delay of all developmental domains. Kept in an incubator for more than two days was a predictor of the delay in all developmental domains except daily living skills. History of any health problem during pregnancy was a predictor for both the FMD (AOR = 1.6, 95% CI:1.0–2.5) and communication delay (AOR = 1.5, 95% CI:1.2–1.9). Neonatal history of cyanosis after birth were likely to have GMD (AOR = 3.4, 95% CI:1.43–7.9) and/or FMD (AOR = 2.3, 95% CI:1.1–4.8) and/or daily living skills delay (AOR = 2.02, 95% CI:1.12–3.6).

Belonging to middle social class was also a predictor for daily living skills delay (AOR = 1.4, 95% CI:1.1–1.7) and communication delay (AOR = 1.3, 95% CI:1.1–1.5). Living in urban communities carried significantly higher odds for only communication delay than living in rural communities (AOR = 1.20, 95% CI:1.0–1.4).

Whereas higher maternal education significantly decreased the odds to have any domain delay by nearly 50% for all domains except for GM. The highest influence was mainly for FM (AOR = 0.29, 95% CI:0.14–0.58). Less paternal education acted as a predictor for both communication (AOR = 1.60, 95%CI:1.3–2.1) and socialization delays (AOR = 1.80, 95% CI:1.1–3.0).

Living in Frontiers decrease the odds to have both daily living skills delay and communication delay nearly by 39% and 65% respectively (AOR = 0.61, 95% CI:0.39–0.95 and AOR = 0.35, 95% CI: 0.2–0.46 respectively) than living in cities. Living in lower Egypt decreases the odds to have daily living skills delay, communication delay, and socialization domain by nearly 35% (AOR = 0.69, 95% CI:0.49–0.98, AOR = 0.59, 95% CI:0.49–0.71& AOR = 0.60, 95% CI:0.41–0.88respectively) than living in cities. Whereas living in upper Egypt decreases the odds to have all delays except for GMD than living in cities.

Strengthening life skills is a popular strategy aiming to resolve problems emerging early in life and promote the health of school children. The spectrum of life skills depends on the age of children and factors affecting their development. Identification of school-aged children with DDs who are at risk for lifelong impairments can improve long-term outcomes through the provision of effective life skills development. Thus, the current cross-sectional national community-based survey was conducted to estimate the national prevalence of DDs among Egyptian children aged 6 to 12 years and their determinants out of the socio-demographic, epidemiological characteristics, and maternal and child medical history.

The results of the present study showed that 7.4% of school-aged children were detected to be developmentally delayed. Those with multiple delays were 3.9% versus 3.5% with one developmental delay (DD). The majority of the studies, especially those in the Middle East and Arab countries and even in Egypt focused on identifying the prevalence of developmental delays among preschool children [39–43]. According to the World Health Organization (WHO), about 5% of the world’s children 14 years of age and under have a moderate to severe developmental disability [39]. Furthermore, most of the Egyptian studies focused on identifying also risk factors of one or more domains of developmental delays among infants, toddlers, and preschool children to act early and prevent later developmental delays [41–43]. Meanwhile, the current study was aiming at screening for DDs among school-age children for providing the baseline data on the delay prevalence of each domain and its associated determinants, as a prerequisite to enhancing the implementation of competent life skills programs and preventing lifetime disability.

The current study showed that 6.4% of school-aged Egyptian children were detected to have a communication delay as the most common type of delay. Language delay is one of the most frequent developmental disabilities in pediatric populations to the extent that up to 5%-15% of the children have delayed language development [44, 45]. Students with language or communication delays have deficits in problem-solving skills and interpersonal relations, so they thought lower than their peers. These children will be at risk of depression, low self-esteem, loneliness, and educational fall. They aren’t accepted by peers and will be neglected [46]. So, LSE programs is suggested to work on these points in order to raise children’s self-esteem. This could be done through speech therapy sessions and programs designed for the participation of schoolteachers, peers as well as parents.

The second domain following communication deficits was daily living skills delay presented by 2%, followed by socialization delay by 1.6%. The development of socialization competencies has important consequences for young children, including the ability to make and maintain quality friendships, and peer acceptance. Young children with social competencies are more likely to experience positive early school adjustment and academic success [47]. Early identification of school-age children with delayed social competencies and behavioral problems provides an opportunity to focus on these problems, and guide the LSE instructor to implement strategies linking parents, classroom teachers, together with peers, to remediate these problems before affecting the academic and social behaviors of the children at school [48].

Good motor abilities are considered essential for children’s physical, social, and mental development. While gross motor skills involve running and jumping, fine motor skills are used for writing and drawing, and both fine and gross motor developmental delays will affect academic performance and may lead to social problems when a child is compared to his/her peers [49]. In the current study, the prevalence of fine motor delay was 1% and the gross motor delay was 0.6%. This prevalence is considerably low if compared to that of other developing countries. Karel et al., 2021 found that the prevalence of fine motor delay in vulnerable preschool children from low-income communities in Australia was 10.4% [50]. Veldman and coworkers, 2020 reported that 4.4% of the children from childcare services had delayed gross motor skills [51]. Both genetic and environmental factors are expected to affect the child motor skills [52]. Prior research has shown that outdoor environments are stimulating and motivating for children to play, climbing, running, jumping and also control of objects [53]. Egyptian children may have better motor skills by genetic predisposition for movement and by being encouraged to get outside and play, especially in rural areas. In addition, lack of easy transportation vehicles helps them to walk, and lack of technology helps them to move [54].

Regarding the risk factors affecting development, the present study showed that belonging to the middle social class and /or living in urban communities carried significantly high odds for DDs. This result is in contrast to other studies. Ozkan et al., 2012 found that low-level household income was strongly correlated with developmental delay in early childhood [55]. Gunardi et al., 2019 also delineated that low education and low income were significant risk factors for DD [56]. Moreover, living in urban communities in the current study carried significantly higher odds of delay for communication and socialization domains. Indeed, different studies [55–59] showed the link between rural communities and DD which is contrary to the current result. They attributed this link with rural areas to differences in demographic patterns, greater financial difficulties, and less access to amenities and treatment resources [57]. However, the present finding for the association of delay with urban communities and middle social class can be attributed to the time lasting long on smartphones, electronic tablets, and television among these communities since a positive relationship had been shown between the early onset and high frequency of TV viewing and communication delay [60]. Living in overcrowded urban environment, causes chronic exposure to air pollution and noise pollution from transportation and industry. Particulate matter or heavy metals, can penetrate the respiratory system via inhalation, causing various diseases including central nervous system dysfunctions which may affect social and communication development [61]. In addition, noise is related to raised catecholamine secretion, modifies social behavior, impairs reading, comprehension and long-term memory in children [62].

Living in frontiers significantly decreased the odds of delay than living in cities for daily living skills by 40% and for communication deficits by 62%, the results could be explained by a decrease in the risk of exposure to pollution together with exposure to nature at frontiers. Nature engagement provides a lot of psychological and physiological benefits, many of which begin in childhood and continue into adulthood, among these benefits are improved cognitive performance, improved sleep and reduced stress [63]. Also living in extended families at frontiers where grandparents, sisters, and brothers live with the parents and their children, each member in the family provides support and protection for the children who grow psychologically competent.

Along the same lines, results showed that living in Upper or Lower Egypt was protective against DD compared to living in cities. This finding could be related to socio-cultural factors influencing acquisition of adaptive behaviour [64]. These localities (Upper and Lower Egypt) are characterized by involvement of children with the whole family in agricultural activities and manufacturing projects which continuously maintains a relationship with the children giving them a higher level of independence and more chances to experience daily living skills [65]. In addition, the extended families, neighbours and expanded social networks may help the children by leading, teaching, providing, caring, and communicating. On the other hand, children in cities lack these opportunities.

In the current study, the male gender was identified as a risk factor for any type of developmental delay. This finding is consistent in many previous studies [66, 67]. This risk associated with the male gender could be attributed to the fact that boys are more likely to be born prematurely, and more vulnerable to neonatal illnesses than are girls [68]. In addition, gonadal steroid hormones trigger different immunological and neural development in boys and girls. A high level of prenatal testosterone not only decreases neuronal development in the left hemisphere in boys but also increases cognitive anomalies in the right hemisphere such as dyslexia and stuttering [69].

Our results showed that a history of maternal health problems during pregnancy or difficult labor increased the probability of delay in both domains of communication and daily living skills. An Iraqi study by Torabi et al., 2012 [58] investigated the correlation between high-risk pregnancy and DD and found a prevalence of 18.7% developmental delay among the group of a high-risk pregnancy with a significant correlation with low birth weight and maternal medical disorders in pregnancy. Moreover, a significant correlation between high-risk pregnancy and the fine motor delay was found. Children who were the outcome of any high-risk pregnancy should be considered suitable candidates for LSE and must be identified early on school entry through detailed medical history documents filled by the parents.

The current results also delineated the risk of prematurity, neonatal convulsion, cyanosis, jaundice, meningitis, and neonatal incubation for more than two days, for the delay in all the studied domains of development. Perinatal risk factors could interfere with normal brain development early in life and may trigger developmental delays in preschool children. Children with early developmental delay commonly demonstrate persistent and steadily poor performance across all developmental and functional domains during the early school years [70]. In agreement, Kerstjens et al., 2011 [71] delineated that at preschool age, the prevalence of developmental delay was higher among early preterm infants than in moderately preterm infants and full-term infants. Sachdeva et al., 2010 [72] similarly found that both prematurity and a history of seizures are biological predictors of global developmental delay. Egyptian Intervention that targeted future mothers for improving the quality of perinatal care improved birth outcomes [73, 74] indicating the value of managing high-risk pregnancies in the prevention of DDs.

The current study showed that the high school education of both parents significantly acted as a protective factor against all types of DDs. This may be attributed to physical care and psychological support provided by the highly educated parents to their children, providing a high quality of stimulation, parallel to engagement in other social activities and in sports. Also, higher education gives the opportunity to work in a distinguished position that generates a high income which may be reflected in their children. Many types of research confirmed the positive effect of paternal education on the domains of child development [66, 75–77].

The age of mothers at giving birth above 35 years significantly increased the odds of daily living skills delay and communication delay than mothers giving birth at a younger age, this may be attributed to biological disadvantages during pregnancy and childbirth. The highest risk of developmental vulnerabilities for mothers aged above 35 years was largely explained by socioeconomic disadvantage [78].

Whereas living without mothers carried almost three times the odds of delay in the socialization domain and in the daily living skills domain, living without fathers carries odds of delay for all the studied domains by nearly twice the presence of fathers. Terry-Ann et al., 2008 found that cognitive outcomes are statistically similar, for children in stable single-parent and stable two-parent households. However, disruptive family structures, characterized by a father’s partial presence in the home, are shown to have adverse effects on cognitive performance compared to the stable single-parent family structure [79]. This study supports the idea that the family stability is the core element in producing positive child outcomes relative to family structure. So, it is important to raise the awareness of parents about the adverse effects of disruptive family structure on their children, as well as recruit these children into a comprehensive program to overcome this problem.

### Strengths

This is the first study in Egypt to address the prevalence and risk factors of developmental delay domains at a national level among School-aged children. The developmental data provided from the current national population study carry high reliability and accuracy for these developmental domains being done on large sample size and representative of the whole country. Our findings highlighted nearly all the important key risks as well as the protective factors which are the cornerstone upon which the implementation of life skills programs could be achieved in an effective and efficient way. Allowing a better understanding of the community under the study and facilitating identifying the situation of DDs domains with their determinants will lay a platform for the nationwide implementation of life skills programs in a cost-effective way. The suggested methodology if approached will accelerate the implementation of life skills programs in the following years by resource-limited countries.

### Limitation

This study had some limitations. First, the study did not investigate the common etiologies of DDs eg. genetic or vascular or toxins, or maternal mental health factors. Second, although there are documented links between growth and development with quality of diet [80, 81] and proven positive effects of dietary supplements or improving dietary behaviors on the health [82], the cognitive, and academic achievements [83, 84] among Egyptian children, this study did not assess the nutritional influence. Lack of assessment of psychosocial problems acts as another limitation. The time factor for conducting these assessments was behind the study limitation.

## Conclusions and recommendations

The present study provided a national estimate for the delay in five developmental domains among Egyptian school children aged 6–12 years. The most affected domain was communication. The study also revealed the sociodemographic and epidemiological characteristics of children who had developmental delays. The study identified a lot of modifiable maternal and child risk factors for DDs. These results are expected to be helpful for more effectively identifying Egyptian school-age children with potential disorders in the clinical setting. The study revealed the protective effect of maternal and paternal education and the role of living with both parents.

Based on the findings of the current study, a set of recommendations are suggested including: focusing on education to raise parental education level and enhancing their awareness about normal and delayed child development, applying vigilant ante-natal and perinatal care as well as screening of children for DD at school entrance. Approaching at-risk children especially those belonging to the middle social class in urban communities- through tailored life skills development programs to raise the quality of their future life and productivity is also recommended. Special emphasis should be directed to studying the influence of the psychosocial problems on children so that LSE programs be directed to raise the student’s communication and social skills if possible.

## Supporting information

S1 Table Targeted households (HH). (DOC)

S2 Table Raw data: Delays data 6–12. (XLS)

## List of abbreviations

LSE: Life Skills Education
DDs: developmental delays
GMD: gross motor delay
FMD: fine motor delay
CAPMS: Central Agency for Public Mobilization and Statistics
UNFPA: United Nations Fund for Population Activities, formerly the United Nations Fund for Population Activities
VABS: Vineland Adaptive Behaviour Scales
MOHP: Ministry of Health and Population
CDC: Cairo Demographic Centre
CI: Confidence intervals
OR: odds ratio
WHO: World Health Organization
LBW: low birth weight
ETPP: Early Truancy prevention project
