# Supplementary material for: National screening for developmental delays and their determinants among Egyptian school age children: A step towards implementing life skills programs
Source: PLoS One. 2023 Sep 19;18(9):e0287315. doi: 10.1371/journal.pone.0287315 (PMC10508607; doi:10.1371/journal.pone.0287315)
Supplement: S1 Table — (DOC) [file pone.0287315.s001.doc]

**S Table-1: List of the targeted Households (HH) according to the governorates, locality and sociodemographic status for screening of DD among children aged 6-12 years**

| serial | **Gov.** | | **classification according to HDI** | **Hai** | **Kesm/Markaz** | **Urban** | | | **Rural** | | | | | Total |  | | | |
| --- | --- | --- | --- | --- | --- | --- | --- | --- | --- | --- | --- | --- | --- | --- | --- | --- | --- | --- |
|  |  |  |  |  |  | **Shiakha (English)** | **Shiakha (Arabic)** | **HH** | **Local Unit ( (English )** | **Local Unit (Arabic)** | **Village (English )** | **Village (Arabic)** | **HH** | **HH** |  | | | |
| **1** | **Cairo** | | **High** | **AlNozhah** | **النزهة** | **Al Hicksit** | **الهايكسيت** | **1390** |  |  |  |  |  | 1390 |  | | | |
|  |  |  | **middle** | **AlSaiedah Zainab** | **السيدة زينب** | **Alkabsh** | **الكبش** | **1390** |  |  |  |  |  | 1390 |  | | | |
|  |  |  | **low** | **AlSharabia** | **الشرابية** | **Al Amiria** | **الأميرية** | **1390** |  |  |  |  |  | 1390 |  | | | |
| **2** | **Dakhlya** | | **High** | **AlSenbelawin** | **السنبلاوين** | **Al Sinblaween city** | **مدينة السنبلاوين** | **317** | **Kafr Alruwk** | **كفر الروك** | **Alshalaa** | **الشعالة** | **905** | 1222 |  | | | |
|  |  |  | **middle** | **MietSalsil** | **ميت سلسيل** | **Mit salsil city** | **مدينة ميت سلسيل** | **317** | **Alatihad** | **الاتحاد** | **AlJafara** | **الجاعفرة** | **905** | 1222 |  | | | |
|  |  |  | **low** | **AlMataria** | **المطرية** | **Almataria city** | **مدينة المطرية** | **317** | **Alsafra** | **العصافرة** | **Al Dahear** | **الضهير** | **905** | 1222 |  | | | |
| **3** | **Gharbia** | | **High** | **KafrElZaiat** | **كفر الزيات** | **KafrElZaiat** | **كفر الزيات** | **260** | **Kafour Belshay** | **كفور بلشاي** | **Qasta** | **قسطا** | **680** | 940 |  | | | |
|  |  |  | **middle** | **Samanood** | **سمنود** | **Samanood** | **سمنود** | **260** | **Ziyad’s locality** | **محلة زياد** | **Munshat Nzif** | **منشأة نظيف** | **680** | 940 |  | | | |
|  |  |  | **Low** | **Markaz of Qutour** | **قطور** | **Qutour** | **قطور** | **260** | **Kotour** | **قطور** | **Khabata** | **خباطة** | **680** | 940 |  | | | |
| **4** | **Fayoum** | | **High** | **Markaz of Al Fayoum** | **الفيوم** | **Alqism rabie** | **القسم رابع** | **145** | **Dacia** | **دسيا** | **Al Sunbat** | **السنباط** | **580** | 725 |  | | | |
|  |  |  | **middle** | **Markaz of**  **Senoures** | **سنورس** | **Senoures** | **سنورس** | **145** | **Terrsa** | **ترسا** | **Alzawia El Khadra** | **الزاوية الخضراء** | **580** | 725 |  | | | |
|  |  |  | **Low** | **Markaz of Tamiaha** | **طامية** | **Tamiaha** | **طامية** | **145** | **Sarsna** | **سرسنا** | **Kafr Omira** | **كفر عميرة** | **580** | 725 |  | | | |
| **5** | **Assuit** | | **High** | **Hay Shark** | **حي شرق** | **Alwalidia Alwustania** | **الوليدية الوسطانية** | **235** | **Bani Hussein** | **بني حسين** | **Musriea** | **مسرع** | **795** | 1030 |  | | | |
|  |  |  | **middle** | **Al Kousiah** | **القوصية** | **Al Kousiah City** | **القوصية** | **235** | **Mir** | **مير** | **Bani Hilal** | **بني هلال** | **795** | 1030 |  | | | |
|  |  |  | **Low** | **Al Ghanaiem** | **الغنايم** | **Al Ghanaiem** | **الغنايم** | **235** | **Alazayiza** | **العزايزة** | **Al Amri** | **العامري** | **795** | 1030 |  | | | |
| **6** | **Aswan** | | **High** | **Nasr Al Nouba** | **نصر النوية** | **Nasr Al Nouba City** | **نصر النوية** | **280** | **Korta** | **قورتة** | **Garf Hussein** | **جرف حسين** | **410** | 690 |  | | | |
|  |  |  | **middle** | **Edfo** | **أدفو** | **Al-Busaliya Bahri** | **البصيلية بحري** | **280** | **Alramad Albahry** | **الرماد بحري** | **Adfu Quabli** | **أدفو قبلي** | **410** | 690 |  | | | |
|  |  |  | **Low** | **Markaz KoomOmbo** | **كوم أمبو** | **KoomOmbo** | **كوم أمبو** | **280** | **Al Abbasia** | **العباسية** | **Sabaa Quabli** | **سبعة قبلي** | **410** | 690 |  | | | |
| **7** | **Damietta** | | **High** | **AlRawda** | **الروضة** | **AlRawda** | **الروضة** | **275** | **Hajaja Village** | **قرية حجاجة** | **Hajaja Village** | **قرية حجاجة** | **380** | 655 |  | | | |
|  |  |  | **middle** | **Al zarqaa** | **الزرقا** | **Alsarw** | **السرو** | **275** | **Sharmsah** | **شرمساح** | **Kafr Toqaa** | **كفر تقي** | **380** | 655 |  | | | |
|  |  |  | **Low** | **Kafr-Saad** | **كفر سعد** | **Kafer Albatiykh** | **كفر البطيخ** | **275** | **Kafr Saad Country** | **كفر سعد البلد** | **Nawasiriya village** | **قرية النواصرية** | **380** | 655 |  | | | |
| **8** | **MarsaMatrouh** | | **High** | **Marsa Matrouh** | **مرسي مطروح** | **MarsaMatrouh**  **(Alsanusia & Kilo 4)** | **مرسي مطروح**  **(السنوسية وكيلو 4)** | **470** | **Alkasr** | **القصر** | **Alkasr** | **القصر** | **220** | 690 |  | | | |
|  |  |  | **middle** | **Al Hamam** | **الحمام** | **Al Hamam City** | **الحمام (النهوض)** | **470** | **Alsalam** | **السلام** | **Alsalam** | **السلام** | **220** | 690 |  | | | |
|  |  |  | **Low** | **AlNajyla** | **النجيلة** | **AlNajyla** | **مدينة النجيلة** | **470** | **Almathany** | **المثاني** | **Almathany** | **المثاني** | **220** | 690 |  | | | |
| **Total** | |  | | | | | | **10116** |  |  |  | | **11910** | **22026** |  |  |  |  |
